# Digital footprints as a new translational approach for mental health care: a commentary

**DOI:** 10.1007/s44192-023-00032-7

**Published:** 2023-01-30

**Authors:** Julio Licinio, Ma-Li Wong

**Affiliations:** Precision Medicine Laboratory in Psychiatry (PMLP), Institute for Human Performance, State University of New York, Upstate Medical University, 505 Irving Ave 3302, Syracuse, NY 13210 USA

## Abstract

There is a crisis in mental health care, with more people suffering from psychiatric disorders than resources that are available for treatment, even though spending is substantial. Millions who suffer from addiction, psychosis, depression and suicidality are either untreated or inadequately treated and organized psychiatry is unable to reach them. Possibly as reflection of under-treatment of psychiatric disorders, the rates of suicide have risen: from 1999 through 2014, the age-adjusted suicide rate in the US increased 24%, from 10.5 to 13.0 per 100,000. Assessment of psychiatric symptoms in ongoing outpatient settings is costly, inadequate and unable to detect clinical changes over time. One’s digital phenotype is assessed through footprints left over as result of our interface with technology, including automated assessments of quantity and quality of social media activity, patterns and speed of device usage, and physiological data that is automatically collected, such as location, quantity and type of movement, heart rate, and sleep patterns. The use of digital footprints has been advocated for large-scale data collection that can facilitate psychiatric research in naturalistic settings. We highlight recent papers in *Discover Mental Health* addressing digital approaches to mental health and we also advance here the concept that digital footprints are ready for clinical use. However, before that happens there needs to be discussion on the appropriate boundaries between care that is driven by signals from digital footprints and the rights to privacy and self-determination.

There is a crisis in mental health care, with more people suffering from psychiatric disorders than resources that are available for treatment, even though spending is substantial. Roehrig reported that in 2013 mental disorders topped the list of most expensive conditions, costing $201 billion [1]. Yet millions who suffer from addiction, psychosis, depression and suicidality are either untreated or inadequately treated and organized psychiatry is unable to reach them [2]. Suicide is highly associated with mental disorders through a general psychopathology factor that represents a shared effect across all mental disorders [3]. Possibly as reflection of under-treatment of psychiatric disorders, the rates of suicide have risen: from 1999 through 2014, the age-adjusted suicide rate in the US increased 24%, from 10.5 to 13.0 per 100,000 [4]. Suicide in now the 10^th^ cause of death [4].

Novel approaches are needed for mental health care. Three recent papers in *Discover Mental Health* have addressed advances in digital approaches to improve mental health. Shen et al. have studied the effects of virtual reality intervention on future self-continuity and delayed reward preference in substance use disorder recovery. Their pilot study results demonstrate the feasibility of an immersive VR intervention designed to increase valuation of the future by enhancing future self-continuity and leveraging future self-discrepancy with personalized future selves as SUD recovery support [5]. An international South American partnership, reported by Cavero et al. addressed issues related to the implementation and scalability of a digital intervention to reduce depressive symptoms in people with diabetes, hypertension or both in Brazil and Peru, with a focus a qualitative study of the perspectives of stakeholders in those health systems [6].

We advance here the concept of using digital tools to improve mental health by proposing here that digital phenotyping obtained through digital footprints may increase access, quality and outcomes of psychiatric care, while at the same time noting there are still important limitations and precautions that need to be taken into consideration. These are distinct from digital biomarkers perspective, which have been described as a more direct (or narrow) concept of signals that are directly linked to biological variables, such as stemming from molecular genetics, epigenetics, endocrinology, immunology or brain imaging, to name a few [7].

When one considers online or device-based approaches to health, including mental health, it is possible to separate those in two broad categories: interactive applications (apps) and digital footprints. Interactive apps can upon request, that is immediate or pre-programmed, deliver questions, surveys, or standardized questionnaires and through this active interface determine the presence and severity of symptoms. There are myriad apps available to track physical and mental health [8–11]. As those require active engagement, they represent an additional step in the ongoing effort to engage subjects into their own treatment. In contrast, digital phenotyping through digital footprints represents a conceptually novel approach to psychiatric symptoms.

The term “digital phenotyping” was introduced by Jain et al. as an extension of Dawkins’s concept of “extended phenotype,” defined as aspects of our interface with technology that can be diagnostic and prognostic for certain conditions [12]. One’s digital phenotype is assessed through footprints left over as result of our interface with technology, including automated assessments of quantity and quality of social media activity, patterns and speed of device usage, and physiological data that is automatically collected, such as location, quantity and type of movement, heart rate, and sleep patterns. The use of digital footprints has been advocated for large-scale data collection that can facilitate psychiatric research in naturalistic settings [13, 14]. We advance here the concept that digital footprints are ready for clinical use.

Assessment of psychiatric symptoms in ongoing outpatient settings is costly, inadequate and unable to detect clinical changes over time [15]. This is not surprising as symptom assessment occurs over increasingly infrequent and brief consultations that are artificially established at pre-arranged times and away from naturalistic settings that may be key triggers for symptomatology. Moreover, self-report and recollection are notoriously inaccurate and biased by the subjects’ mental status. Self-reports are affected not only by the state of mind, but also by the social desirability of the reported behavior, as well as by the ceiling effects of artificially constructed rating scales [16]. In contrast, digital footprints provide data that are inexpensive, abundant, and collected nearly continuously, in the context of the individual’s natural environment and interpersonal activities.

There are ongoing efforts to validate the precision of digital footprints as proxies for established neuropsychological parameters. Dagum has compared standard neuropsychological assessment to data from digital footprints [17]. For several neuropsychological constructs (working memory, memory, executive function, language and intelligence) it was determined that a family of digital biomarkers predicted test scores with highly significant correlations. Those results suggest that passive measures from smartphone use may become a continuous ecological surrogate for laboratory-based neuropsychological assessment. An elegant demonstration of the use of passive data to assess depressive symptoms naturalistically was provided by Saeb et al. who showed that mobile phone location sensor data are inversely related to depressive symptom severity over a 10-week period, during which global positioning system (GPS) phone sensor data was compared to depressive symptoms [18]. Ongoing studies are now adding digital footprints for mood assessments: Madrid et al. have included digital footprint-based measures of anhedonia in an ongoing trial of a novel type of antidepressant, BTRX-246040, a selective antagonist of the human nociceptin receptor [19]. Markowetz et al. included in a longitudinal study of depression severity digital footprints such as (i) app usage, (ii) social interaction, and (iii) macro-movement (assessed through GPS) [14].

We typically think of translation as going from the bench to the bedside. However, a new modality of translation may bring naturalistic big data from ubiquitous devices to improve mental health care. As evidence accumulates on the validity of using digital footprints for mental health research, are we ready to translate them into psychiatric care? Dr. Thomas Insel, former Director of the National Institute of Mental (NIMH) believes so [20, 21]. He left the NIMH and is now leads start ups in Silicon Valley aimed at utilizing digital phenotyping in mental health. Google and other companies have similar initiatives.

It is critical to use digital phenotyping to address suicide risk. Christensen et al. have suggested that technology can be helpful by combining the power of big data analysis with information gathered digitally at the time of risk in order to identify individual-level suicide risk profiles that can be provided to individuals (within ethical frameworks) and clinicians [22].

The promise of inexpensive, naturalistically collected data that can longitudinally provide information on key symptoms, responses to treatment, and suicide risk could revolutionize mental health care and go beyond that. Behavior, cognition, and emotional states are relevant to the management of chronic diseases, such as diabetes, asthma and arthritis. However, a multitude of implementation and ethical quagmires emerge as one translates such data to day-to-day mental health care. Bauer et al. discussed ethical perspectives related to the use of digital technology in psychiatry [23]. Pitfalls include the potential for medical harm related to poor quality online information, self-diagnosis, which may be highly inaccurate, leading to the dangers of self-treatment, and the limitations of passive monitoring. Devices commonly used to collect digital footprints were not created, validated, and certified for clinical assessments. Moreover, who will clinically validate the enormity of big data generated from digital footprints into clinically useful guidelines? It is unlikely that government funding will support such research that is not driven to test novel hypotheses. Industry has immense bias, as it will directly profit from the algorithms that it will test and validate.

A complex ethical quandary will undoubtedly emerge from privacy related to digital footprint monitoring. After assessment of signals, subjects or their treatment providers may be electronically warned that there is clinical deterioration—and may be risk for suicide—yet said patients may refuse to seek care [24]. After a threshold of severity is reached, health care providers may be put in the awkward position of being liable if they do not deliver in person interventions for those at high risk, but they may also be liable if they deliver unnecessary or involuntary interventions. A new debate is needed among stakeholders, such as patients, families, providers, institutions, industry, government agencies, and bioethicists as to the appropriate boundaries between emergency care that is driven by signals from digital footprints and the rights to privacy and self-determination [25].

## References

[1] Roehrig C (2016). Mental disorders top the list of the most costly conditions In the United States: $201 Billion. Health Aff.

[2] Licinio J (2004). A leadership crisis in American psychiatry. Mol Psychiatry.

[3] Hoertel N, Franco S, Wall MM, Oquendo MA, Kerridge BT, Limosin F (2015). Mental disorders and risk of suicide attempt: a national prospective study. Mol Psychiatry.

[4] Curtin SC, Warner M, Hedegaard H (2016). Increase in suicide in the United States, 1999–2014. NCHS Data Brief.

[5] Shen YI, Nelson AJ, Oberlin BG (2022). Virtual reality intervention effects on future self-continuity and delayed reward preference in substance use disorder recovery: pilot study results. Discover Mental Health.

[6] Cavero V, Toyama M, Castro H, Couto MT, Brandt L, Quayle J (2022). Implementation and scalability of a digital intervention to reduce depressive symptoms in people with diabetes, hypertension or both in Brazil and Peru: a qualitative study of health system’s stakeholders’ perspectives. Discover Mental Health..

[7] Montag C, Elhai JD, Dagum P (2021). On blurry boundaries when defining digital biomarkers: how much biology needs to be in a digital biomarker?. Front Psychiatry.

[8] Chan S, Godwin H, Gonzalez A, Yellowlees PM, Hilty DM (2017). Review of use and integration of mobile apps into psychiatric treatments. Curr Psychiatry Rep.

[9] Hui CY, Walton R, McKinstry B, Jackson T, Parker R, Pinnock H (2017). The use of mobile applications to support self-management for people with asthma: a systematic review of controlled studies to identify features associated with clinical effectiveness and adherence. JAMIA.

[10] McMillan KA, Kirk A, Hewitt A, MacRury S (2017). A Systematic and integrated review of mobile-based technology to promote active lifestyles in people with type 2 diabetes. J Diabetes Sci Technol.

[11] Cornet VP, Holden RJ (2018). Systematic review of smartphone-based passive sensing for health and wellbeing. J Biomed Inform.

[12] Jain SH, Powers BW, Hawkins JB, Brownstein JS (2015). The digital phenotype. Nat Biotechnol.

[13] Bidargaddi N, Musiat P, Makinen VP, Ermes M, Schrader G, Licinio J (2017). Digital footprints: facilitating large-scale environmental psychiatric research in naturalistic settings through data from everyday technologies. Mol Psychiatry.

[14] Markowetz A, Blaszkiewicz K, Montag C, Switala C, Schlaepfer TE (2014). Psycho-informatics: big data shaping modern psychometrics. Med Hypotheses.

[15] Hatfield D, McCullough L, Frantz SH, Krieger K (2010). Do we know when our clients get worse? An investigation of therapists' ability to detect negative client change. Clin Psychol Psychother.

[16] Katschnig H (2006). Quality of life in mental disorders: challenges for research and clinical practice. World Psychiatry.

[17] Dagum P. Digital biomarkers of cognitive function. NPJ Digital Medicine. 2018 [in press]. 10.1038/s41746-018-0018-4 PMC6550173 31304295

[18] Saeb S, Lattie EG, Schueller SM, Kording KP, Mohr DC (2016). The relationship between mobile phone location sensor data and depressive symptom severity. PeerJ.

[19] Madrid A, Smith D, Alvarez-Horine S, Saljooqi K, Dagum P, Mahableshwarkar A (2017). Assessing anhedonia with quantitative tasks, digital and patient reported measures in a multicenter, double-blind trial with BTRX-246040 for the treatment of major depressive disorder. Neuropsychopharmacology.

[20] Insel TR (2017). Join the disruptors of health science. Nature.

[21] Insel TR (2017). Digital phenotyping: technology for a new science of behavior. JAMA.

[22] Christensen H, Cuijpers P, Reynolds CF (2016). Changing the direction of suicide prevention research: a necessity for true population impact. JAMA Psychiat.

[23] Bauer M, Glenn T, Monteith S, Bauer R, Whybrow PC, Geddes J (2017). Ethical perspectives on recommending digital technology for patients with mental illness. Int J Bipolar Disord.

[24] Berrouiguet S, Barrigon ML, Castroman JL, Courtet P, Artes-Rodriguez A, Baca-Garcia E (2019). Combining mobile-health (mHealth) and artificial intelligence (AI) methods to avoid suicide attempts: the Smartcrises study protocol. BMC Psychiatry.

[25] Kim A, Hsu M, Koire A, Baum ML (2022). Incidental findings from deep phenotyping research in psychiatry: legal and ethical considerations. Camb Q Healthc Ethics.

